# Towards a shared understanding of sustainability for neglected tropical disease programs

**DOI:** 10.1371/journal.pntd.0009595

**Published:** 2021-08-20

**Authors:** Jeffrey Glenn, Aparna Barua Adams, Girija Sankar, Carolyn Henry, Karen Palacio, Wangeci Thuo, Katherine Williams

**Affiliations:** 1 Sustainable Systems Cross-Cutting Group, Neglected Tropical Disease NGO Network, London, United Kingdom; 2 Department of Public Health, College of Life Sciences, Brigham Young University, Provo, Utah, United States of America; 3 International Coalition for Trachoma Control, London, United Kingdom; 4 CBM International, Bensheim, Germany; 5 SCI Foundation, London, United Kingdom; 6 The END Fund, New York City, New York, United States of America; 7 RTI International, Washington, DC, United States of America; Swiss Tropical and Public Health Institute, SWITZERLAND

## Abstract

**Background:**

Sustainability within neglected tropical disease (NTD) programs is a complex and challenging issue. The need for a shared understanding about what sustainability means for NTD programs is more important than ever as stakeholders are currently realigning for the next decade of NTD programming with the launch of WHO’s new NTD roadmap for 2012–2030. The aim of this paper is to assess different perspectives to generate a working definition of sustainability for NTD programs.

**Methodology/Principal findings:**

This study surveyed affiliates of the NTD NGO Network (NNN) about their definitions of sustainability and then analyzed the data using an inductive and deductive process. The research team drafted a sustainability statement based on the survey findings and then solicited and incorporated feedback on the statement from a diverse group of expert reviewers. The final statement includes a working definition of sustainability for NTD programs that highlights three key essential components to sustainability: domestic commitment, responsive resource mobilization, and accountability.

**Conclusions/Significance:**

This research resulted in a sustainability statement, based on a survey and extensive consultation with stakeholders, that represents a starting point for shared understanding around the concept of sustainability for NTD programs. Future collaborative work should build off this definition and seek to incorporate indicators for sustainability into programmatic decision-making.

## Author summary

The question of whether a global public health program can be sustainable is as important as whether that program is effective. While neglected tropical disease (NTD) programs have achieved tangible success in reducing the burden of NTDs over the past decade through a massive collaboration between global and local stakeholders, the achievement of global NTD control and elimination goals will depend in large part on whether these efforts are sustained. This study seeks to encourage more and better dialogue around NTD program sustainability by incorporating a wide variety of expert perspectives to create and propose a shared definition of sustainability upon which future NTD programming decisions can be made. The sustainability statement based on the findings from this study suggests that three essential components to NTD program sustainability are commitment from decision-makers within NTD-endemic countries, resource mobilization that is responsive to local needs, and enhanced mechanisms for accountability.

## Introduction

Over the past decade, neglected tropical diseases (NTDs) have increased in prominence as a global health priority while experiencing immense progress towards ambitious shared goals, established in the 2012 World Health Organization (WHO) roadmap for NTD control, elimination, and eradication [1]. Forty countries have now eliminated at least one NTD, and 500 million fewer people no longer require interventions against NTDs compared to 2012 [2]. Yet, there remains a critical need to focus on the long-term sustainability of NTD programs, their impact, and their contribution to building resilient health systems. NTD-focused organizations currently lack a shared definition of sustainability, resulting in disparate ideas about what the concept means and how it should be measured and operationalized to achieve a world free of NTDs. The need for a shared understanding about what sustainability means for NTDs is more important than ever as stakeholders mobilize for the next decade of NTD programming with the launch of WHO’s new NTD roadmap for 2021–2030 [2].

The term “neglected tropical diseases” (NTDs) encompasses 20 bacterial, viral, and parasitic diseases that are found most commonly in tropical and subtropical environments [3]. These diseases continue to represent a major disease burden in many parts of the world, particularly among the 2.7 billion people living on less than US$2 per day [4]. Together, these diseases are estimated to cause approximately 350,000 deaths and are responsible for 27 million disability-adjusted life years annually [5]. The focus of global NTD control and elimination efforts has largely been on the five “PC-NTDs”—lymphatic filariasis, onchocerciasis, soil-transmitted helminthiases, schistosomiasis, and trachoma—that can be prevented through preventive chemotherapy (PC) medicines distributed via mass drug administration (MDA) [6]. Control and elimination of these five NTDs is widely seen as one of the best investments in global health, with effective MDA interventions in many circumstances costing less than US$.50 per person per year [5,6]. Despite significant success in expanding access to PC drugs for people with NTDs, serious concerns exist regarding whether endemic countries’ MDA programs can continue without significant support from international development partners and donors [7]. Additionally, a focus within these programs on procuring and distributing donated drugs has made it harder for national NTD programs and partners to advocate for other activities such as behavior change communication or water and sanitation (WASH) interventions [8,9,10]

*Ending the neglect to attain the Sustainable Development Goals*: *a sustainability framework for action against neglected tropical diseases 2021–2030*, a document published in 2021 by WHO, sets out global targets and actions to align and re-focus the work of endemic countries, partners and stakeholders during the next decade, including cross-cutting targets aligned with the Sustainable Development Goals [2]. Country ownership, sustainability, and cross-sectoral partnership all lie at the very heart of this new road map, recognizing that achieving these ambitious global NTD goals will require a paradigm shift towards a comprehensive approach to ending NTDs. The COVID-19 pandemic has illustrated the essential need for strengthened national health systems, resilient enough to weather public health crises. Through the economic crises laid bare from the COVID-19 pandemic, we also know that the NTD community must be able to do more with fewer resources, and increasing partnerships across sectors will be critical to the achievement of the new road map, and ultimately support sustainable impacts of NTD programs, delivering on universal health coverage for all. Multi-sectoral interventions across WASH, vector control, disability inclusion and innovative and intensive disease management (IDM) are all essential to controlling, eliminating, and eradicating a range of NTDs, and ensuring their accessibility to all requiring interventions. Sustained progress for all NTDs will depend on prioritization of this wide range of strategies at the policy, programmatic, and financial levels.

One important sign of progress is that NTDs are now widely seen as a critical component of the broader development agenda, as evidenced by their inclusion in the Sustainable Development Goals (SDGs)—NTDs are directly mentioned in SDG target 3.3, which calls to “end the epidemics of … neglected tropical diseases” [11]. Addressing NTDs also plays a critical role in achieving many of the other SDGs—e.g., alleviating poverty and hunger, enabling access to education, promoting equality—and progress towards other SDGs can accelerate the achievement of NTD goals—e.g., provision of clean water and sanitation, resilient infrastructure, and climate action [2].

Program sustainability is a complex concept for which a number of different perspectives exist in the academic literature. Although the details of various definitions and frameworks differ, at its core, sustainability typically refers to the continuation of program activities and benefits over time [12,13]. Sustainability can be considered in terms of its determinants–i.e., the programmatic, organizational, and contextual factors that lead to a continuation of program activities–as well as its outcomes–i.e., the degree to which the program is sustained as measured by dose, reach, and fidelity of the continued activities. Some scholars argue that, since sustaining any global health program over time will require continual adjustments to inevitable contextual change, sustainability should be viewed as a dynamic process of adaptation rather than a goal in itself [14,15].

While sustainability of NTD programs has typically received less attention for diseases with elimination goals, most experts recognize the need for a sustainable NTD response integrated into strong local health systems [16,17,18,19,20]. A number of important factors may contribute to a sustainable response to NTDs. The WHO and others have long called for increased country ownership—a major area of emphasis in WHO’s new NTD roadmap—and less reliance on external donor funding for NTD programs as a means of promoting sustainability [2,7,21,22,23,24]. In recent years, endemic countries and global stakeholders have increased their collective efforts to build the capacities of local health teams to independently manage their own programs and to procure medicines locally [3,25]. Community participation is considered an important component of sustainable approaches [26,27,28]. Sustainability also requires multiple approaches beyond preventive chemotherapy, including vector control, WASH infrastructure, ongoing education and communication, and continued surveillance [7,29,30,31,32].

In September 2017, the NTD NGO Network (NNN) established the Sustainable Systems Cross-Cutting Group to assist the global NTD NGO community in establishing and operationalizing a shared understanding of sustainability to promote collaborative approaches for sustainable control and elimination of NTDs. The NNN is a global forum for NGOs dedicated to NTD control, elimination, and eradication efforts. Since 2009, the NNN has provided a common platform for implementing partners and academic institutions to align program goals and activities in support of national NTD programs, guided by the World Health Organization. This study represents the initial efforts of the NNN Sustainable Systems Cross-Cutting Group to assess potential differences in perspectives and generate a clear working definition of sustainability for NTD programs.

## Methods

The primary method of data collection for this article was an electronic survey distributed to affiliates of the NNN. In addition, further consultations with health ministry officials and WHO regional offices were conducted to solicit feedback about the survey findings. Survey questionnaire items included open-ended questions asking respondents to provide their definition of sustainability for NTD programs, to list up to three key phrases or words that characterize sustainability, and to identify challenges to achieving sustainability. The survey gave respondents the option to list their name and asked them to indicate the type of organization with which they are affiliated, their geographic area of focus, and their disease area of focus within NTDs.

The online survey was sent via email in June 2018 to all members and observers (composed of WHO, donor agencies, health ministry officials, and private sector organizations) of the NNN. The membership body of the NNN includes national and international NTD-focused non-governmental organizations as well as academic institutions. Members of the NNN Sustainability Cross-Cutting Group sent reminder emails to specific individuals and encouraged attendees of the September 2018 NNN annual conference to participate in the survey. Participants gave their implied consent by agreeing to participate in the survey. The survey closed at the end of September 2018. Members of the Cross-Cutting Group then analyzed the data by generating basic descriptive statistics of respondents and conducting a thematic analysis, using a deductive approach, of survey responses to identify themes. The study was approved by the Brigham Young University Institutional Review Board (IRB#: IRB2021-057).

After the data collection period concluded, members of the NNN Sustainability Cross-Cutting Group reviewed the survey responses and themes with a focus group at the NNN conference in Addis Ababa, Ethiopia in September 2018. A smaller group then drafted a statement that contained a working definition of sustainability based on the most commonly emerging key concepts.

Because survey respondents primarily represented NGOs and academic organizations, the Cross-Cutting Group sought additional input by sharing this definition with and requesting feedback from 18 reviewers who work on NTD programs within the WHO or NTD-endemic countries. Five of the reviewers were from WHO headquarters or WHO regional offices, and the rest were from national NTD programs in sub-Saharan Africa (8), southeast Asia (3), or south Asia (2). Reviewers were nominated by Cross-Cutting Group Members, and their feedback was discussed and incorporated into the final NNN statement on sustainability. Those who responded had minor feedback but were largely supportive of the working definition as originally written. The Cross-Cutting Group coordinated their work with WHO and with disease coalitions engaged in similar sustainability-focused discussions to ensure efforts would be aligned and not duplicative.

## Results

### Survey findings

Table 1 lists the affiliations, geographic focus areas, and disease focus areas of survey respondents. Of the 113 individuals who were sent the online survey, 49 completed it for an overall response rate of 43%. Of these, the majority (82%) were affiliated with a non-governmental organization. The remaining respondents were from academia (14%), government (2%), and a UN organization (2%), which is consistent with the overall composition of individuals associated with the NNN. In terms of geographic area of focus, 80% of respondents reported a focus on Africa, 22% on Asia, 12% on the Americas, and 8% on the global level. Respondents’ specific disease focus areas within NTDs was concentrated on the five PC-NTDs: 51% on STH, 49% on schistosomiasis, 33% on LF, 20% on trachoma, and 14% on onchocerciasis. Additionally, 20% of respondents mentioned leprosy as a major area of focus. Six of the other NTDs were mentioned once as shown in Table 1.

**Table 1:** Respondent Characteristics.

	Number (N = 49)	Percentage
**Affiliation**		
Non-Governmental	40	82%
Academia	7	14%
Government	1	2%
UN Organization	1	2%
**Geographic Focus**		
Africa	39	80%
Asia	11	22%
Americas	6	12%
Global	4	8%
**Disease Focus**		
Soil-Transmitted Helminthiases	25	51%
Schistosomiasis	24	49%
Lymphatic Filariasis	16	33%
Trachoma	10	20%
Leprosy	10	20%
Onchocerciasis	7	14%
Chagas	1	2%
Echinococcosis	1	2%
Leishmaniasis	1	2%
Podoconiosis	1	2%
Yaws	1	2%

When asked to define sustainability in the context of NTD programs, respondents brought up clear and common themes that align closely with elements of sustainability found in the literature. The top response, mentioned by 47% of survey respondents, highlights the idea that sustainability involves NTD-endemic countries making decisions about and managing their own national programs. Closely related to this, 29% responded that sustainability requires endemic countries to provide funding for their own self-sufficient programs, and 24% mentioned that it requires the NTD program benefit to occur without external funding. The remaining top themes all touched on the continuation of program activities and benefits. Sixteen percent simply stated that in a sustainable program the program benefit, including disease elimination and long-term management, is maintained over time, while 14% mentioned that program activities must continue at a high level of quality. Twenty percent of respondents specified that sustainability entails the maintenance of NTD services by the country health system.

Respondents were also asked to narrow their definitions by listing up to three key phrases or words that characterize sustainability to them. The top theme identified by the research team from these responses, mentioned by 59% of respondents, focused on the idea of local ownership. Other important elements included domestic funding (23%), integration with other NTD programs and within health systems (23%), longevity (20%), and capacity building (18%). Consistency, accountability, and efficiency also received multiple mentions as important concepts that characterize sustainability.

Finally, another key question asked respondents to identify some of the challenges of achieving sustainability for NTD programs. Although this item prompted a wider variety of responses than the previous questions, a few common challenges emerged, with limited available funding being the top response (21%). A number of responses focused on challenges within endemic country national and local governments: low priority of NTDs (17%), lack of political commitment (10%), limited capacity (10%) and lack of government buy-in (8%). Adding a different perspective, some responses highlighted challenges with program funders: donor-driven priorities different from local needs (13%), vertical programming (10%), reliance of countries on external funders (8%), ineffective coordination mechanisms between programs (8%), and a top-down development mindset (6%). Additional responses mentioned the lack of a shared definition of sustainability, the difficulty inherent in multisectoral action, and the need for transparency and accountability.

### NNN statement on sustainability

After carefully reviewing the survey responses along with other resources related to sustainability for NTD programs [7,30,33,34,35], the Cross-Cutting Group drafted a working definition of sustainability. This definition was shared with a wider audience of 18 reviewers for feedback through the consultative process described above. Reviewers agreed with the need to create a shared definition of sustainability and were supportive of the Cross-Cutting Group’s draft. After making slight modifications to the definition to reflect reviewer feedback that emphasized the importance of domestic political commitment, the Cross-Cutting Group presented it at the NNN annual conference in September 2019 where it was officially endorsed by the NNN Executive Committee and Chair. The final version (https://www.ntd-ngonetwork.org/sustainability-a-statement-from-the-nnn) reads as follows:

Sustainability, for NTD programming, is realized when the intended result is achieved for as long as required. Sustainability is not a binary achievement that either exists or doesn’t; rather, it is a spectrum comprised of multiple context-specific factors.

A central theme of sustainability is a national government’s long-term commitment to a goal or result with multi-level, local ownership. Sustainability and ownership are further characterized by the three components of domestic commitment, resource mobilization, and accountability. The three components are closely linked and may not always be completely independent of one another.

1. **Domestic commitment** to NTD programming is multifaceted: it requires high-level political will and dynamic leadership at national, regional, and sub-regional levels. An effective, impactful program will ideally be driven by community demand for continued services and may have shared goals with health systems strengthening and universal health coverage efforts (i.e., the Sustainable Development Goal 3).
2. **Responsive resource mobilization** means that countries are engaged in determining and mobilizing the inputs required to reach their NTD goals and are taking initiative to quantify and fill the resource gaps. Sustainable resourcing involves leveraging domestic investments alongside partner contributions, as local ownership tends to be limited when a program is financially dependent on external sources.
3. **Accountability** is outcome-oriented, coupled with task-oriented responsibility to deliver high-quality results.\

## Discussion

The most important output of this study is the development of the NNN statement on sustainability presented above, which was developed through a survey and consultative process with a wide range of stakeholders from the global NTD community. This statement serves the purpose of creating a starting point for shared understanding around the concept of sustainability for NTD programs. While there are likely individuals and organizations within the NTD community that take a different perspective on aspects of the statement, this shared reference is valuable in allowing stakeholders to identify particular points of agreement and disagreement, and then to enter a more productive discussion articulating their similar and differing approaches. Given the need for a stronger focus on long-term impacts of NTD investments after control, elimination, and eradication targets have been reached, this shared understanding is also essential for collaborative efforts to create and measure sustainability for NTD programming. This paper and the NNN statement on sustainability are not arguing that NTD programs should go on forever; rather, there is an urgent need for a clear understanding of sustainability to ensure the impact of NTD investments will achieve NTD goals and be long-lasting for the health system after NTD elimination goals have been reached.

**Fig 1:**
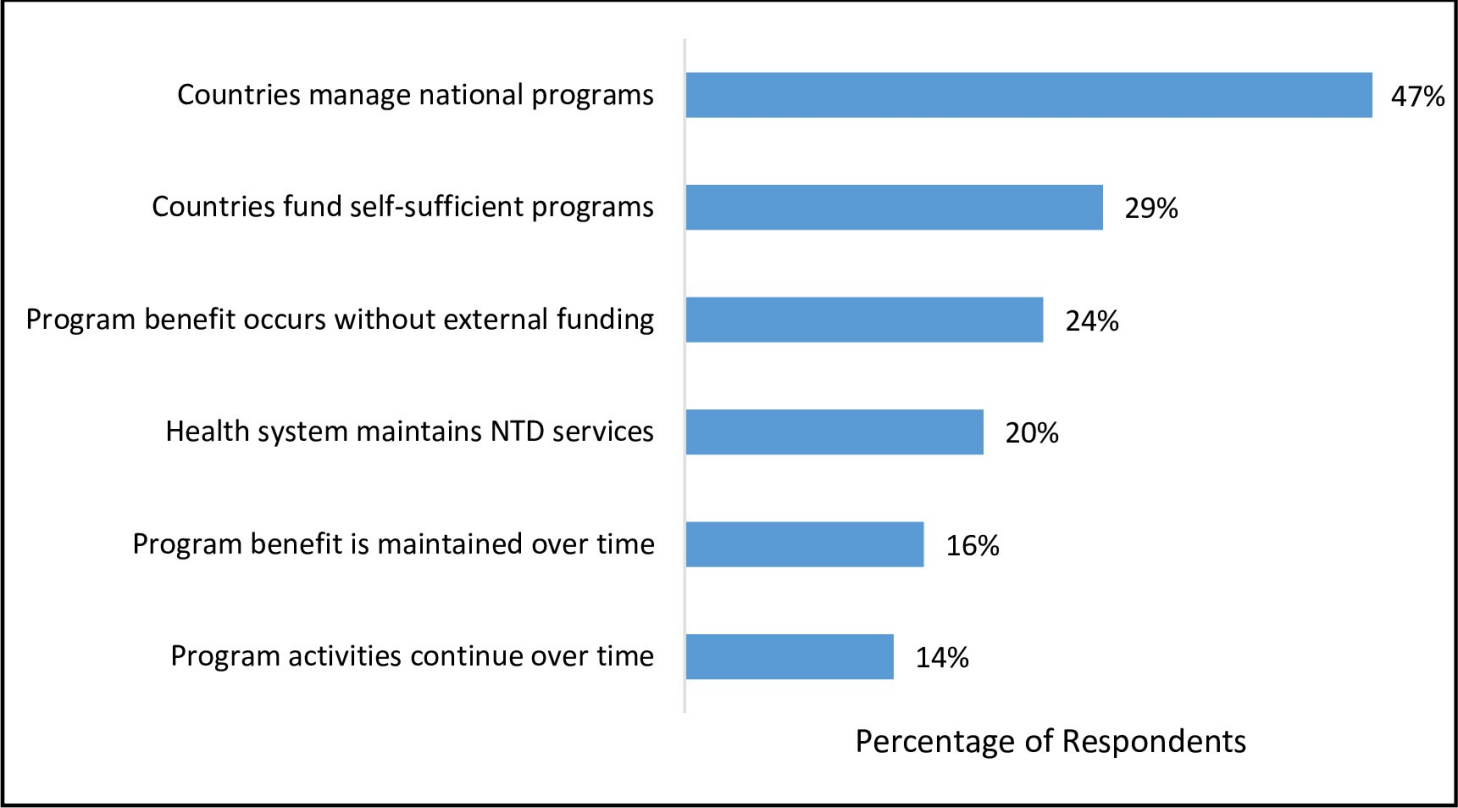
Elements Mentioned in Definitions of Sustainability.

**Fig 2:**
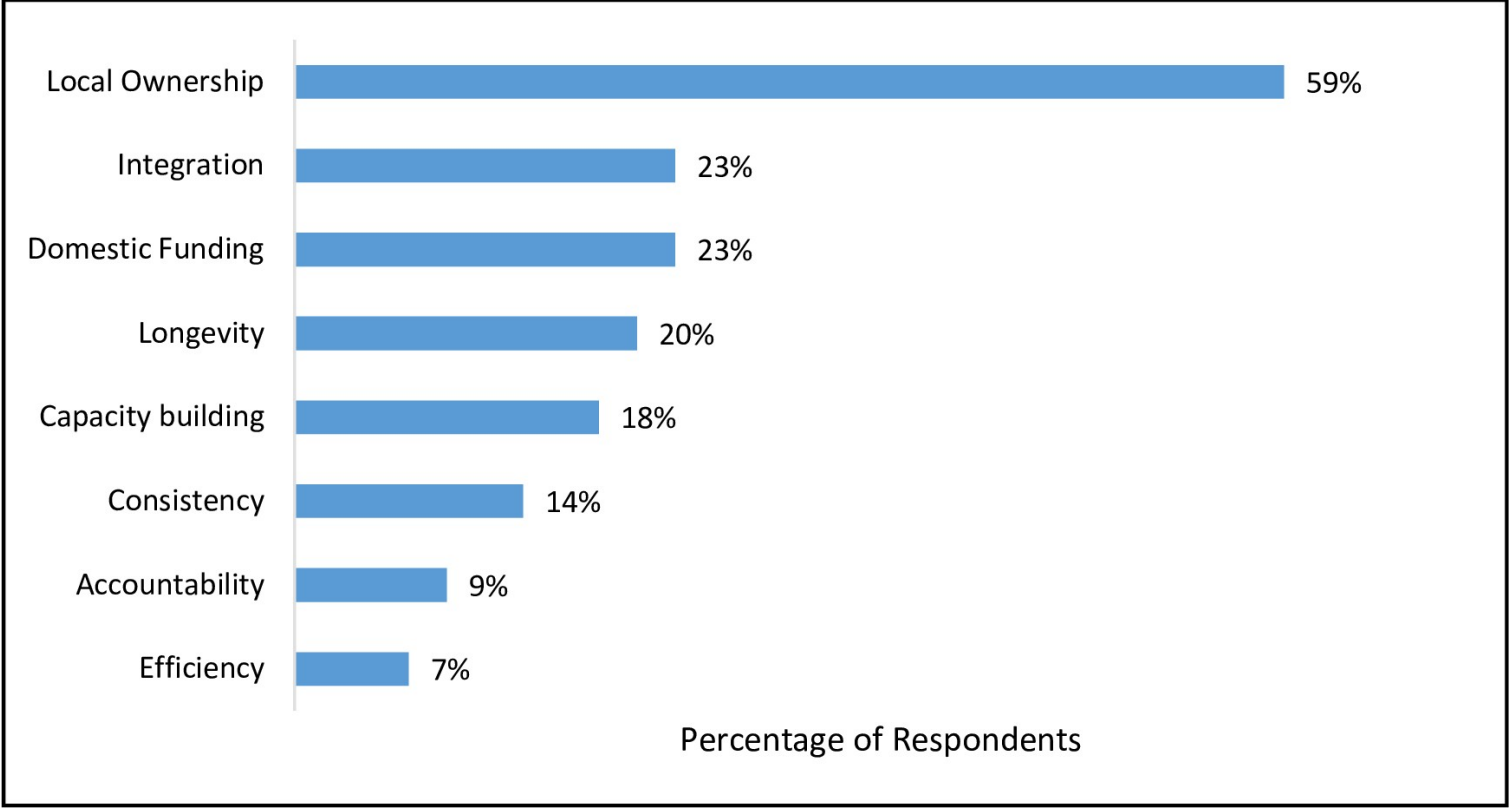
Themes of Words and Phrases Mentioned for Characterizing Sustainability.

**Fig 3:**
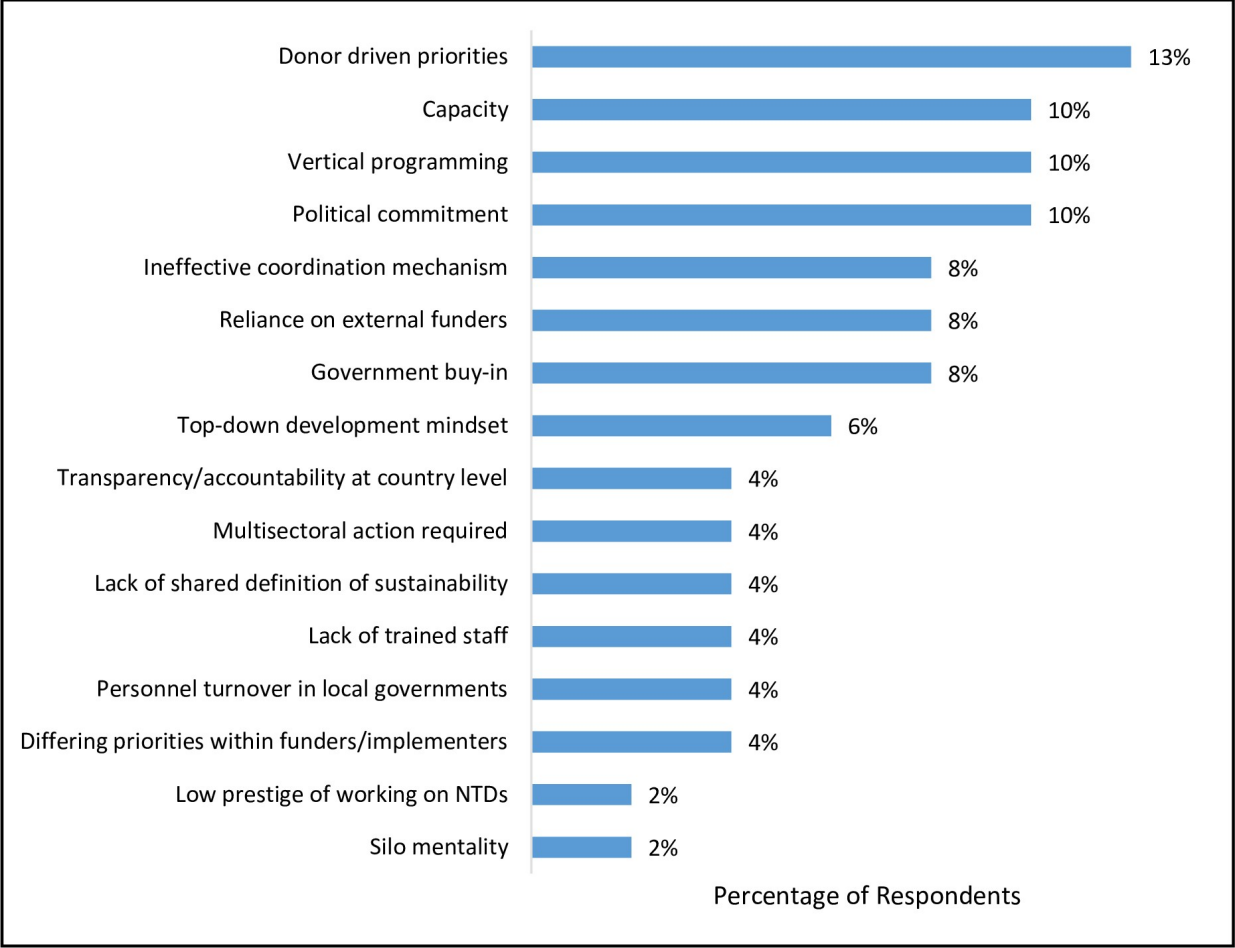
Challenges Mentioned for Achieving Sustainability.

Key elements highlighted in the survey results align with important factors in the sustainability literature and have been incorporated into the NNN statement as central components of sustainability. Given that the top four themes (Fig 1) in respondents’ definitions of sustainability and the top three themes (Fig 2) in respondents’ reported words that characterize sustainability dealt with domestic commitment, respondents clearly view this as a critical issue. Many of the challenges mentioned (Fig 3) offer further support that NGO stakeholders see limited country ownership as the biggest barrier to achieving lasting impact. While obviously not a blind spot for the global NTD community given the amount of attention this issue has received in the academic and gray literature, successfully addressing this complex challenge is proving difficult [7,22,23]. The updated WHO NTD roadmap for 2021–2030 sets a new paradigm, within the context of the broader sustainability agenda for development, for considering the next phase of action to address NTDs [2]. This roadmap emphasizes the need for more extensive country ownership and offers useful guidance that this approach must be supported by organizational structures, leadership that extends from the national to the local levels and across sectors, and a shift in thinking and culture around NTDs.

Enhancing domestic commitment to NTD programming requires stakeholders at all levels to adopt new approaches to achieving key elements of sustainability (local ownership, domestic financing, integration, capacity building, etc.) mentioned by survey respondents. In practice, there are many cases of progress towards increased domestic commitment to NTDs. For example, the END Fund, a global private philanthropic initiative, has dedicated significant financial resources to strengthening local ownership of NTD programs in Nigeria by supporting the development of local champions who have the capacity to help policymakers better understand community needs [36]. In Uganda, the Ministry of Health recently launched its own NTD Program Sustainability Plan 2020–2025 which lays out how NTD priorities will be integrated into routine government planning and health system strengthening investments [37]. The government of Uganda also announced its commitment to increase national funding for NTDs from 12% to 30% by 2025.

Several respondents identified gaps in capacity building as challenges to enhancing domestic commitment to move towards sustainability. To support capacity building within the NTD sector, the WHO has developed the NTD Programme Manager training course [38]. This program has been delivered in Ethiopia since 2016 and involves formal coaching and peer to peer exchange of knowledge designed to increase management skills of junior staff by connecting with experienced mentors. Despite the progress being made towards domestic commitment in many circumstances, these efforts require sustained resources and take time to have the desired effects. In NTD drug supply chain management, drugs for PC-NTDs are received through vertical donation programs. Recent assessments to address this capacity gap found that integrating NTD supply chains into national health supply chain networks contributes to building in-country supply chain capacity, strengthens integration with other NTD and health sectors, improves forecasting of medicine demand, and most importantly, improves access to and demand for NTD services [39].

A closely related element to domestic commitment highlighted in the survey and in the NNN statement is that of resource mobilization. Survey respondents mentioned concerns about the total level of funding being insufficient and about the source of funding being primarily external aid. Some respondents perceived “donor-driven” programs or a “top-down development mindsets” were major barriers to sustainable progress. Thus, the NNN statement on sustainability emphasizes resource mobilization that is responsive to local needs and driven by country program leaders in order to facilitate domestic ownership of programs and outcomes. Since the reality is that donor aid will remain a critical funding source for NTD programs for the foreseeable future, funding mechanisms fixed obligation grants shift decision making power to local governments and allow funds to flow as close to the NTD-endemic communities as possible [40]. In the longer-term, it will be critical to reduce NTD programs’ dependence on foreign aid by securing domestic government funding and by creating innovative global financing mechanisms for NTDs that, for example, may be similar in structure to the International Finance Facility for Immunisation that provides funds to Gavi through issuing Vaccine Bonds to investors [41].

Finally, accountability and transparency were themes that respondents raised as important components of and challenges to achieving sustainability. Since accountability is dependent on NTD programs being able to demonstrate their delivery of high-quality results, the availability of reliable data is crucial to sustainable NTD programs. While this still represents a major challenge in many cases, progress towards increased accountability is being seen with the implementation of District Health Information System V.2 (DHIS2)—a flexible open-source information system being widely used for collecting, analyzing and sharing NTD data [3]. For example, NTD programs in Ethiopia, Kenya, and Malawi have reported success in using DHIS to increase reporting and accurately track program outcomes [42].

Despite the survey and extensive consultative process undertaken to arrive at the sustainability statement, the final definition primarily reflects the views of the NGO sector responsible for coordinating the funding and implementation of local NTD programs. While the statement serves as a valuable starting point, it should be considered a working definition, and additional research should more explicitly seek input from more diverse branches of the global NTD community, including national policy and decision makers as well as local government officials. Future research on this topic should build use more in-depth qualitative methods to understand how perspectives differ by region, disease focus, etc.

The NNN statement on sustainability presented in this paper aims to be a useful tool for NTD NGOs, working alongside and supporting NTD-endemic countries in the implementation of the WHO Sustainability Framework for Action against NTDs, which was launched in early 2021 as a companion tool to the new road map [2]. While the findings included in the NNN statement were not necessarily surprising, the process of gathering data across NNN stakeholders and analyzing the findings within the Cross-Cutting Group is an important step towards sustainable NTD programs. Bringing organizations together to share expertise and opinions has been beneficial in increasing collective understanding of the nuances of sustainability and in setting the foundations for future shared work. The statement alone will fail to generate progress towards sustainable progress until it is operationalized by those who hold responsibility for funding and implementing NTD programs. An important next step is to agree upon program indicators for sustainability, based on the statement and the WHO sustainability framework, and to work collectively to incorporate these indicators into programmatic decision-making criteria.
